# Hybrid MM/SVM structural sensors for stochastic sequential data

**DOI:** 10.1186/1471-2105-9-S9-S12

**Published:** 2008-08-12

**Authors:** Brian Roux, Stephen Winters-Hilt

**Affiliations:** 1 Department of Computer Science, University of New Orleans, LA, 70148, USA; 2 Research Institute for Children, Children's Hospital, New Orleans, LA, 70148, USA

## Abstract

In this paper we present preliminary results stemming from a novel application of Markov Models and Support Vector Machines to splice site classification of Intron-Exon and Exon-Intron (5' and 3') splice sites. We present the use of Markov based statistical methods, in a log likelihood discriminator framework, to create a non-summed, fixed-length, feature vector for SVM-based classification. We also explore the use of Shannon-entropy based analysis for automated identification of minimal-size models (where smaller models have known information loss according to the specified Shannon entropy representation). We evaluate a variety of kernels and kernel parameters in the classification effort. We present results of the algorithms for splice-site datasets consisting of sequences from a variety of species for comparison.

## Introduction and background

We are exploring hybrid methods where Markov-based statistical profiles, in a log likelihood discriminator framework, are used to create a fixed-length feature vector for Support Vector Machine (SVM) based classification. The core idea of the method is that whenever a log likelihood discriminator can be constructed for classification on stochastic sequential data, an alternative discriminator can be constructed by 'lifting' the log likelihood components into a feature vector description for classification by SVM. Thus, the feature vector uses the individual log likelihood components obtained in the standard log likelihood classification effort, the individual-observation log odds ratios, and 'vectorizes' them rather than sums them. The individual-observation log odds ratios are themselves constructed from positionally defined Markov Models (pMM's), so what results is a pMM/SVM sensor method. This method may have utility in a number of areas of stochastic sequential analysis that are being actively researched, including splice-site recognition and other types of gene-structure identification, file recovery in computer forensics ('file carving'), and speech recognition.

We test our pMM/SVM method on an interesting discrimination problem in gene-structure identification: splice-site recognition. In this situation the pMM/SVM approach leads to evaluation of the log odds ratio of an observed stochastic sequence, for splice-site and not, by Chow expansion decomposition, with vectorization rather than sum of the log odds ratios of the conditional probabilities on individual observations (where the conditional probabilities are pMM's, and the odds are on splice-site probability versus not-splice-site probability). By focusing on a particular application of the pMM/SVM method, this also allows us to demonstrate some of the subtleties that occur in implementation, and how they can be resolved by information theoretic criteria, here via use of Shannon Entropy in particular.

Our work makes use of Support Vector Machines for several reasons. Firstly, SVM classifiers have a strong generalized application in machine learning making advances in techniques using them in Bioinformatics directly applicable to other fields utilizing SVM based classifiers. Secondly, the techniques introduced here to automatically target relevant data positions based on entropy analysis have direct contributions to expanding the ability to use SVM classifiers in an unsupervised manner. Finally, though there are existing classifiers currently in use for splice site detection the MM/SVM hybridization is presented here as a novel manner of training against stochastic datasets.

### Shannon Entropy

Shannon Entropy [1] or Information Entropy is a measure of uncertainty or randomness for a given variable in a system. One of the original usages [2] for Shannon entropy was the measure of information conveyed on average for symbols in a given language, and it has significant applications in cryptography and other fields where information content must be quantified. The entropy is calculated as a product of probability and the logarithm of probability for each possible state of the targeted variable. Suppose we have the discrete probability distribution p(x_i_), for the probability of events x_i _for 'i' in [1..N], i.e., p(x_i_) is a discrete probability distribution with N states. Then, Shannon entropy is: -∑p(x_i_)log(p(x_i_)), where the log function in log_2_, ln, or log_10 _results in entropy measured in bit, nat, or dit, respectively. The DNA alphabet, in particular, only has four states: Adenine(A), Cytosine (C), Guanine (G), and Thymine(T), so N = 4 in computations involving this primitive alphabet.

### Splice sites

**Figure 1:**
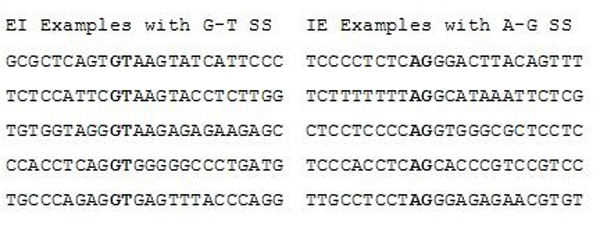
Examples of GT – AG splice site sequences.

Coding regions in eukaryotic DNA are typically interrupted by non-coding regions (95% of cases for protein coding). These non-coding regions are removed by splicing after transcription where pre-mRNA intron segments are removed, and the exon segments remaining are joined together to form the final mRNA. The sequences at the splice region are dominated by GT and AG dinucleotide pairs at the intron side of the Exon-Intron (EI) and Intron-Exon (IE) transitions, respectively (see Fig 1).

### Markov Model

Also known as a Markov Chain, a Markov Model ("MM") is a stochastic process with "short-term" memory. If there were infinite memory, then the probability of observation X_0_, given prior observations {X_∞_,..., X_1_}, would be expressed as P(X_0_|X_∞_...X_1_). In practice, there is neither the data to support such an infinitely detailed conditioning argument, nor the need. (The existence and utility of highly accurate short-term memory representations relate to fundamental aspects of our physical world, such as equations of motion, causality, entropic increase, and equilibration.) For an Nth-order Markov Model (MM) we have: P(X_0_|X_N_...X_1_) [3]. When using MMs part of the model selection problem is the choosing the highest order model that is well-represented by the training data available.

### Positionally-defined Markov Model (pMM)

In the standard Markov analysis of an event X_0_, with prior events {X_N_...X_1_}, i.e. a memory of the past N events, our fundamental mathematical constructs are the conditional probabilities P(X_0_|X_N_...X_1_). For the analysis we describe here we generalize this formalism, further, to also depend on position vis-à-vis some reference point. In the case of splice-site recognition, positionally-defined Markov Models are used to describe event probabilities at various positions on either side of the splice site (also known as a Profile HMM [4]). A pMM is defined as the probability of event X_0_, with Markov order N, at position I: P(X_0_|I; X_N_...X_1_).

### Support Vector Machines

SVMs provide a system for supervised learning which is robust against over training and capable of handling non-separable cases. Learning with structural risk minimization is the central idea behind SVMs, and this is elegantly accomplished by obtaining the separating hyperplane between the binary labeled data sets (± 1) that separates the labeled data sets with a maximum possible margin [5,6]. The power of this approach is greatly extended by the added modeling freedom provided by a choice of kernel. This is related to preprocessing of data to obtain feature vectors, where, for kernels, the features are now mapped to a much higher dimensional space (technically, an infinite-dimensional space in the case of the popular Gaussian Kernel).

**Figure 2:**
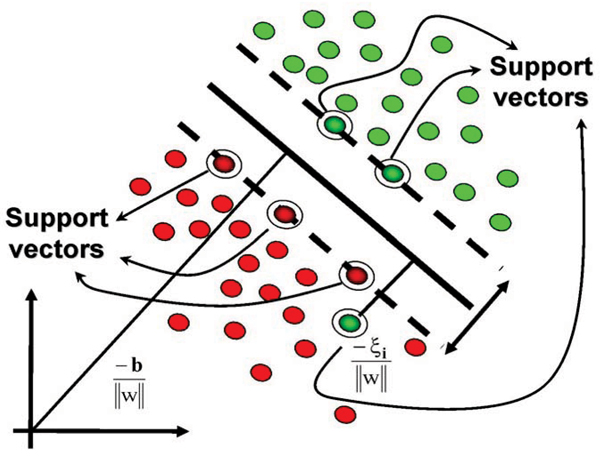
Illustration of a hyperplane separation of two labeled groups in feature space.

The hyperplane itself is centered at **w**·**x **- *b *= 0 where **w **is the normal vector to the separating hyperplane, **x **is the vector of points satisfying the above equation, and *b *is the offset from the origin. Given this, **w **and *b *are chosen to maximize the distance or gap between parallel hyperplanes **w**·**x **- *b *= -1 and **w**·**x **- *b *= 1 (see [7] for more details on the implementation we use). The separable case for the SVM occurs where there is no crossover from the labeled groups over the hyperplane. Non-separable cases are handled through the use of slack values [6] (see Fig. 2) to allow for some cross over in order to still obtain the largest possible margin between the bulk of the labeled groups. One of the strengths of SVMs is that the approach to handling non-separable data is almost identical to that for separable data. Further SVM generalizations, even applications in unsupervised learning/clustering, appear to be possible [7].

Upon introducing Kernels, the SVM equations are solved by eliminating w and b to arrive at the following Lagrangian formulation: max ∑_(i = 1...n) _α_i _- 1/2 ∑_(i, j = 1...n) _α_i _α_j_y_i_y_j _K(x_i_, x_j_), subject to α_i _≥ 0 and ∑_(i = 1...n) _α_i_y_i _= 0, where the decision function is computed as f(x) = sign(∑_(i = 1...n) _α_i_y_i_K(x_i_, x_j_) + b), and where K(x_i_, x_j_) is the kernel generalization to the inner-product term, <x_i_, x_j_>, that is obtained in the standard [6], intuitively geometric, non-kernel based SVM formulation.

## Results

### Shannon entropy analysis

We analyzed large data sets using a variety of MM based techniques to study the areas of lowered entropy within splice site sample sequences. This analysis was critical to identifying information-rich sequence regions around the splice site locations, and are used in defining the positional range of pMM's needed in the SVM classification that follows. We perform an analysis of the 0^th ^order pMM profile of the Shannon entropy delineated splice site regions, then consider the 1^st ^and 2^nd ^order profiles similarly.

We begin by analyzing the Shannon-entropy of the pMM at various orders for the sample sequences, and search for contiguous regions with lower than average entropy which we refer to as the low Entropy ("lEnt") regions. This is the segment of positional data drawn on to generate feature vectors based on pMM data. The initial entropic analysis using the 0^th ^order pMM is used to identify base-positions that have low Shannon entropy. Further analysis using higher order pMMs is used to determine if accounting for greater memory further lowers the entropy of a given position in the sequence. It is found that the positions identified in the lEnt regions carry information about the splice site which a trained SVM can classify with high accuracy.

#### EI 0^th ^Order pMM

**Figure 3:**
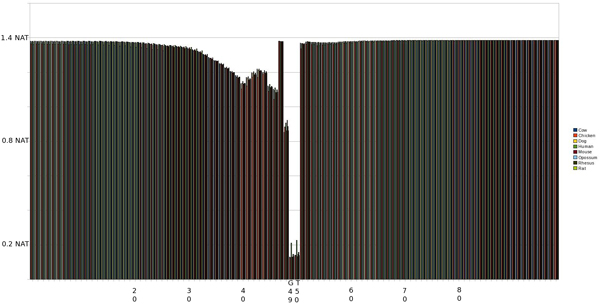
Graph of entropy at each position in the sequence using a 0th Order pMM on an EI SS. The SS occurs at positions 49 and 50.

As shown in Fig. 3, the majority of the exon (right) and intron (left) positions maintain a high level of entropy around 1.4 nat but there is a marked decrease in entropy around positions 49 and 50 which correspond to the splice site (see earlier background for high degree of GT for EI splice sites), as expected. There is a noticeable lEnt region corresponding to the 4 positions on the intron side of the splice site (SS+4) with no lEnt region identified in the exon portion of the sequence (using 0^th^-order pMM's).

#### IE 0^th ^Order pMM

**Figure 4:**
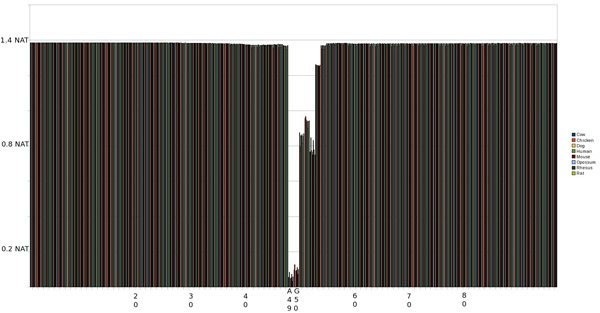
Graph of entropy at each position in the sequence using a 0th Order pMM on an IE SS. The SS occurs at positions 49 and 50.

As shown in Fig. 4, there is a much larger lEnt region in the IE transition, but with a more gradual drop in entropy which is not nearly as pronounced outside of the splice site consensus at positions 49 and 50 (again corresponding to background information). There is also an interesting spike at 2 positions before the splice site (SS-2) at which entropy returns to the normal base line (consistent with what has been noted by biologists).

#### EI pMM 1^st ^& 2^nd ^Order Entropy

**Figure 5:**
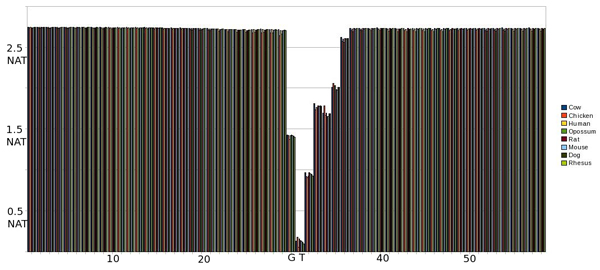
Graph of entropy at each position in the sequence using a 1st Order pMM on an EI SS. The SS occurs at positions 30 and 31.

**Figure 6:**
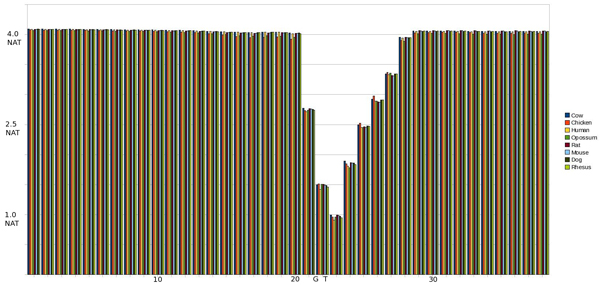
Graph of entropy at each position in the sequence using a 2nd Order pMM on an EI SS. The SS occurs at positions 21 and 22.

With first order pMM on the EI transition we see the entropy on the first splice site residue increase in proportion to surrounding entropy as compared to the MM Profile entropy for EI (see Figs 5 &6). This is indicative of the high entropy for positions near the splice site. Specifically the position preceding the splice site (SS-1) influences the first splice site position and increases entropy. When we extend the EI pMM to 2^nd ^order we observe the entropy increases more evenly the further it extends from the splice site. Additionally we see the lowest entropy point shift further into the intron section under the influence of both residues in the splice site. Along the same lines as the EI 2^nd ^order pMM, IE shows a more gradual transition than 1^st ^order or MM Profile, along with a lessening of the entropy spikes seen previously.

#### IE pMM 1^st ^& 2^nd ^Order Entropy

**Figure 7:**
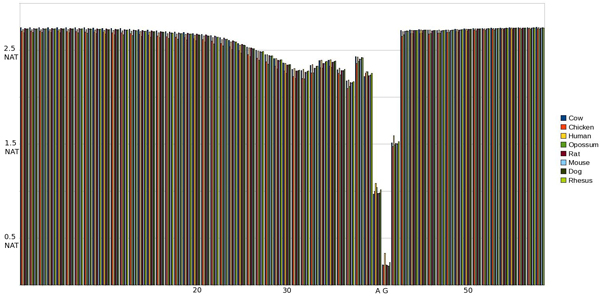
Graph of entropy at each position in the sequence using a 1st Order pMM on an IE SS. The SS occurs at positions 40 and 41.

**Figure 8:**
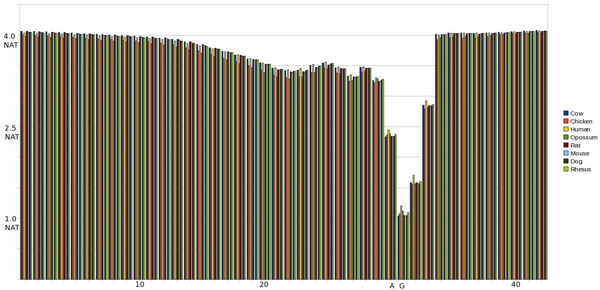
Graph of entropy at each position in the sequence using a 2nd Order pMM on an IE SS. The SS occurs at positions 30 and 31.

A similar result is achieved when analyzing IE splice site sequences under pMM 1^st ^Order (see Figs 7 &8). We note the decrease in entropy from the exon position following the splice site (SS+1) due to the influence of the low entropy splice site residues. Also of note, however, is the entropy spike toward the end of the intron region (SS-2) which becomes lessened when influenced by the surrounding intron residues in the LET Region. Along the same lines as the EI 2^nd ^order pMM, IE shows a more gradual transition than 1^st ^order or MM Profile, along with a lessening of the entropy spikes seen previously.

### Feature extraction, kernel selection, and SVM training

**Figure 9:**
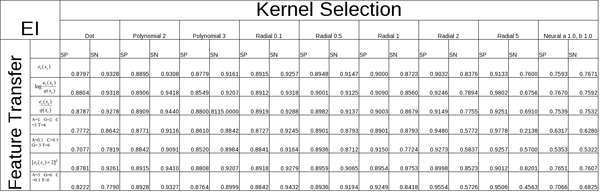
Table overview of results from feature transfer functions (y-axis) and kernel/parameter selections (x-axis) for EI SS samples.

**Figure 10:**
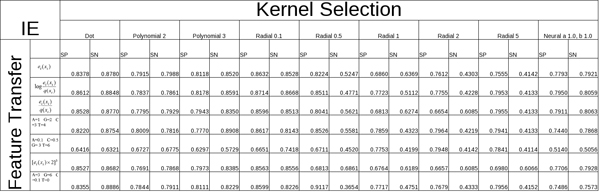
Table overview of results from feature transfer functions (y-axis) and kernel/parameter selections (x-axis) for IE SS samples.

Through feature extraction we translate the nucleic acids in the sequence, along with the information garnered from the pMM at various orders, into a numeric value which we transfer into a vector. This is accomplished using a variety of functions with differing amounts of success as detailed in our results. Other feature vector extractions are used that involve ratios between event probability and background probability, as well as direct symbol to numeric transliterations. It appears a number of feature vector rules can be successful, as shown in the Tables in Figures 9 and 10, in the sense that they can provide the basis for strong SVM classification of splice sites.

Once a feature vector has been produced from the data, by pMM preprocessing in particular, the discriminating task is passed to the SVM. The success of an SVM with a given data set can be greatly improved with a tuning over kernels (and kernel parameters). Efforts to automate this tuning on choice of kernels is currently being explored by use of genetic algorithms (further discussion of that effort is not included here). In the work presented here, we explore a variety of kernels, as shown in the Tables in Figures 9 and 10, including the Dot, Polynomial, Radial, and Neural kernels, where each of the kernels is tuned and scored on its best performing kernel parameters.

In the tables shown in Figs 9 and 10, the SVM performance is shown for various feature extraction methods. The 0^th^-order pMM based method elaborated on here, with log likelihood elements log(e_i_(x_i_)/q(x_i_)), is one of the better performing cases, where e_i_(x_i_) is the pMM for the i^th ^position and q(x_i_) is the generic background probability for observation x_i _(not positionally dependent). For the null case, or negative instances, we select false splice site locations from the true data by choosing positions outside the splice site regions. These feature vectors are split in half, with one set used to train the SVM and the other used to evaluate the SVM's performance (against data it was not trained against). The accuracy is measured in terms of Sensitivity ("SN") and Specificity ("SP"). By comparing the {SN, SP} of the training data to the {SN, SP} of the testing data we can evaluate the SVM's classification performance, where the generalization, "real world", performance is estimated by the scoring with the test data (and an algorithmic probe of the best performance possible is done by testing on the training data).

#### Overview of kernels tested

A variety of methods for feature extraction as well as kernel types and parameters have been tested to see how well the data sets responded to each. The results for these initial tests based on the data sets obtained from [8] are presented in Figs 9 (EI) and 10 (IE), which show 2 dimensional table comparisons, where the Y-axis represents the feature transfer function used, and the X-axis represents the specific Kernel function and parameter(s) selected. The table entries themselves show results for Sensitivity ("SN") and Specificity ("SP"). The Radial Gamma function was chosen to test these results more extensively, along with feature extraction using pMM's.

Results for this are obtained for four species: Cow, Chicken, Human, and Opossum, and are shown in the EI and IE Results that follow.

#### EI splice site results

We use the Radial kernel with gamma set to 0.5, combined with using Log(e(x)/q(x)) where e(x) is the emission probability, and q(x) is the background probability, for a given residue. These results use much larger data sets than initial trials based on data from [8], and show comparison across species boundaries.

**Figure 11:**
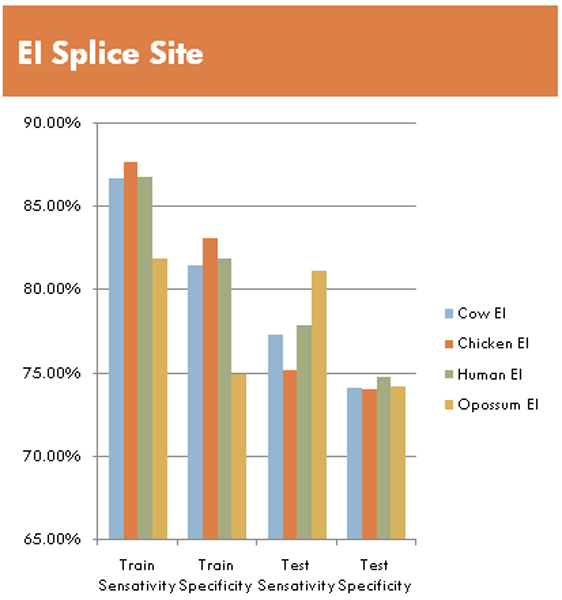
Overview of selected results from the larger multi-species datasets using radial kernel on EI sequences.

Human was chosen as the base line, with Cow selected for evolutionary similarity as a fellow mammal. Chicken was selected for evolutionary distance between itself and human/cow, and Opossum as a marsupial was similarly chosen for its distance from Chicken, and for not being as close to Human as Cow. Figure 11 shows the results from training and testing. Classification on training data has sensitivity ranges from 80% to 90%, and specificity in the 80–83% range, except for Opossum which drops to 75% on specificity. These results give an idea what the best-case performance should be. Actual classification on the test data, for a true estimate of learning generalization performance, is found to have a 10% reduction in sensitivity, and a 5% reduction in specificity when compared to the 'best-case' training data performance. Interestingly, the Opossum results are stable with almost negligible change in accuracy when testing on the train and test data sets. The low training results in EI are likely due to the much smaller feature vector size due to a smaller lEnt region for the 0^th ^order pMM, this is noticeably less in the IE results as we will now examine.

#### IE splice site results

**Figure 12:**
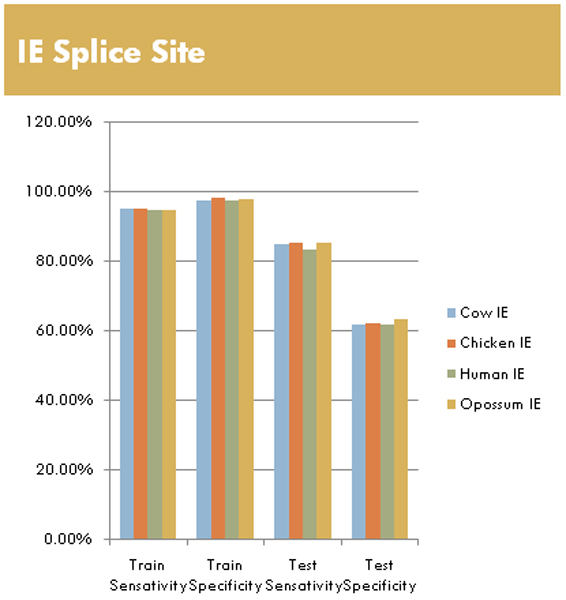
Overview of selected results from the larger multi-species datasets using radial kernel on IE sequences.

The IE feature vector size is much larger (15 vs 4) than the EI size. As such, there is a much more stable training result due to IE's SVM being in 15 dimensional space vs the 4 dimensional space for EI. Results are detailed in Fig. 12, for the same species examined for EI. In comparison to the EI results, both training sensitivity and specificity are close to 100% accuracy. Transitioning to testing gives a drop of approximately 15% for testing sensitivity, but around 40% in specificity (i.e., resulting in 85% SN and 60% SP). Unlike the EI Opossum results, the IE Opossum results on train and test sets are in line with the Cow, Chicken, and Human behavior.

## Conclusion

The main result of this preliminary study shows pMM/SVMs can be trained as splice site classifiers with high accuracy. We believe this approach is applicable to other problem sets, and represents a new approach that combines entropy analysis for feature selection and eventual pMM/SVM classification. From the specific examples shown, we see that the splice-site classification results using the pMM/HMM approach are very promising, for both IE and EI splice sites. By changing from a 0^th ^Order pMM to a higher order pMM, it is possible to extend the low entropy (lEnt) region at the cost of adding noise to the low entropy positions. This increase in the lEnt region allows a lift to an SVM with a higher dimensional feature space, which has an impact on initial training results (as shown in the differences between Figs 11 and 12 with vector size 4 and 15, respectively). In ongoing efforts we hope to work with pMMs of higher order, and to begin training SVMs using the 1^st ^and 2^nd ^Order pMM's. This effort is meant to eventually contribute to ongoing construction of a new gene finder approach (by SWH) that leverages the power of SVMs and MM variations (such as those involving gap interpolating MMs).

## Methods

### pMM/SVM method

In the typical log likelihood discriminator construction, such as for identification of splice sites, binary classification is provided by the sign of the log odds probability of the splice site vs non-splice-site region. The log odds probability, in turn, is obtained from the sum of the log conditional probabilities from the Chow expansion of observing the observed sequence in the splice-site vs non-splice-site models. In the pMM/SVM method, a sum is not produced from the log conditional probabilites, but a vector. The length of the feature vector depends on the number of terms in the Chow expansion, i.e., on the length of sequence used in the splice-site recognition model. For the splice-site recognition problem described here, an SVM-based classifier is explored for a variety of sequence window sizes (4–20 components). The window size is then determined in an automated fashion, that is minimally sized, by use of Shannon entropy analysis of splice-site alignments.

### Shannon entropy data

In our research we use Shannon entropy analysis to identify locations of lowered entropy within the sequence surrounding a splice-site. With this automated process we can identify areas of the sequence with lower entropy. These segments of the sequence are less random and therefore contain more information than the remainder of the splice. Using the feature transfer function we transfer the positions identified by Shannon entropy analysis into a feature vector for classification by SVM.

Initial research utilized a small data set of human splice regions originally extracted from GenBank Rel.123 [8]. This set contains approximately 2,700 true EI and 2,800 true EI sequences combined with with 300,000 IE false and 270,000 EI false sequences. Splitting the dataset evenly into four (EI test, EI train, IE test, IE train) created a fast turn around for training and testing amongst the various SVM kernel definitions and parameters (results shown in Figs 9 and 10).

For more in-depth statistical analysis a larger data set was obtained. Given the resistance of SVMs to over training, we elected to train with a more even ratio of true and false sequence instances. For each species approximately 125,000 true and 125,000 false sequences each for IE and EI, giving a total set of 500,000 sequences for each species between the IE train, IE test, EI train, and EI test sets. Species used for testing include: 1. Chicken; 2. Cow; 3. Dog; 4. Human; 5. Mouse; 6. Opossum; 7. Rat; and 8. Rhesus Monkey.

## Competing interests

The authors declare that they have no competing interests.

## Authors' contributions

SWH conceptualized the project and performed the preliminary pMM/SVM tests. BR performed the extensive Shannon entropy tests, and the pMM/SVM tests with the large multi-species datasets. SWH and BR each contributed to the writing and approved the final manuscript.
